# Frontal Alpha Asymmetry Argues for the Heterogeneity of Psychological Resilience

**DOI:** 10.3390/brainsci13091354

**Published:** 2023-09-21

**Authors:** Christopher F. Sharpley, Ian D. Evans, Vicki Bitsika, Wayne M. Arnold, Emmanuel Jesulola, Linda L. Agnew

**Affiliations:** 1 Brain-Behavior Research Group, University of New England, Armidale, NSW 2350, Australia; ievans3@une.edu.au (I.D.E.); vicki.bitsika@une.edu.au (V.B.); warnold3@myune.edu.au (W.M.A.); doctorseasept@yahoo.com (E.J.);; 2 School of Science & Technology, University of New England, Queen Elizabeth Drive, Armidale, NSW 2351, Australia; 3 Department of Neurosurgery, The Alfred Hospital, Melbourne, VIC 4222, Australia; 4 Griffith University, Nathan, QLD 4222, Australia

**Keywords:** depression, resilience, frontal, alpha, asymmetry

## Abstract

Depression is associated with frontal alpha asymmetry (FAA) and Psychological Resilience (PR), although in different ways. Only cursory attention has been given to how these three constructs interact despite the possible clinical and research implications of those associations. One limitation of recent research into these associations has been conceptualising PR as a unitary construct, whereas it has been shown to be multi-component. This study investigated the underlying components of PR, their correlations with FAA, and the effect that participants’ depressive status had upon those correlations in a community sample of 54 males and 46 females aged between 18 yr and 75 years. Results confirmed the overall inverse association between total PR and depression for four of the original five PR components and for one of the two components found in this sample. Similarly, there were differences between the ways that FAA and PR components were associated, depending upon the depressive status of participants. Source localisation data indicated that the PR components were not uniformly correlated with alpha activity in the same brain regions. These findings of content, efficacy, and neurophysiological differences between the five components of PR and their associations with FAA argue against consideration of PR as a unitary construct.

## 1. Introduction

Depression is a major and widespread disorder [1] for which medication and psychotherapy treatments have only limited efficacy [2,3,4,5]. Consequently, in the search for additional therapy options, it has been argued that attention to patient-centred attitudinal factors such as Psychological Resilience (PR) might be productive [6], particularly as these attitudinal factors are often able to be learnt via training [7]. PR is defined as an individual’s capacity to cope with immediate stressors and avoid the harmful effects of negative events in the future [8]. A great deal of previous literature supports the “buffering” effects of PR upon the depressive outcomes of stressful events, such as those experienced by older people [9], cancer patients [10], and those who experience chronic pain [11], terrorist attack [12], personal violence [13], and even the demands of COVID-19 lockdown [14].

It has been suggested that PR functions by reducing autonomic responses to stressors (It has been suggested that PR functions by reducing autonomic responses to stressors [15], and thereby helps the individual to maintain, or return to, calm when faced with stressors. An example of PR’s calming effect on the autonomic nervous system is the alleviation of sleeping difficulties, which may occur due to hyper-arousal states [16]. As such, PR may be seen as a neurophysiologically based precursor to psychological constructs such as Optimism, which has been shown to be inversely correlated with depression [17]. On this basis, some previous research has focussed upon the neurophysiological correlates of PR, with some of those findings indicating that children who have been maltreated but who demonstrate PR also have greater left frontal brain hemisphere activity than children who are not resilient [18]; a similar finding was reported for persons who had experienced PTSD [19]. Although some recent attention has been given to dynamic processes within the brain that are associated with PR (e.g., flexibility between resting brain networks) [20], the frontal brain asymmetry construct remains of major interest in understanding PR, particularly since one form of asymmetry has been repeatedly associated with a range of psychiatric disorders, including depression (e.g., [21,22,23]). This form of asymmetry is most commonly measured in the alpha wave band (8–13 Hz), and is known as frontal alpha asymmetry (FAA). Alpha wave activity represents the relative absence of brain activity in other frequencies, such as beta waves (e.g., 13 to 18 Hz) which are associated with more intense mental activity such as active concentration, controlling movement, and anxiety [24,25,26]. Alpha activity is thus used as an inverse indicator of other activity in a particular brain site.

The association between FAA and PR may also provide an insight into the neurobiological substrates of PR and how these interact with depression. Several studies have commenced this research, focussing on a variety of EEG-related variables, including brain network flexibility [27], response to transcranial magnetic stimulation [28], and event-related potential reactions to an odd-ball presentation [29], but the greatest concentration of research has been on frontal lobe alpha asymmetry (e.g., [30,31,32,33]). These studies are usually performed with participants who have undergone some form of stress. For example, Bae et al. [30] found that FAA was inversely associated with the quality of recovery after thyroidectomy, and Ma et al. [31] reported that FAA was significantly associated with heart rate and cortisol responses to the Trier Social Stress. Additionally, Meiers et al. [33] examined the association between childhood trauma symptoms and adolescent problem behaviour, finding that FAA moderated the positive association between trauma symptoms and problem behaviour. In a systematic review of this field, Silveira et al. [32] concluded that FAA (which is defined by a higher left than right hemisphere prefrontal cortex alpha activation) was associated with greater participant affect, energetic arousal, lower anxiety, and calmness. Although of value in understanding the association between FAA, stress and related issues, these studies do not shed light on the association between FAA, PR and MDD, leaving that issue open to further investigation.

Underlying the frontal asymmetry-depression hypothesis and potentially valuable in explaining the association between PR, FAA, and depression, it has been suggested that two behavioural systems coordinate adaptive behaviour. These are the Behavioural Inhibition System (BIS), which increases attention towards aversive events, processes potential threats, and prepares the individual for robust withdrawal responses to those threats, and the Behavioural Activation System (BAS), which activates responses to cues of reward and initiates approach-related behaviour [34]. Although some early models have suggested that the BAS is associated with relatively greater left frontal activity than right frontal activity and the reverse for the BIS [35,36,37], some subsequent studies suggest that withdrawal and approach behaviours may both be subsidiaries of the BAS [38]. Other research has found that withdrawal behaviour per se may be associated with left frontal brain activity [39], thus challenging the basic cerebral dichotomy model of the BAS/BIS and approach/withdrawal activity.

Although these models of brain-behaviour correlates are valuable in understanding how PR interreacts with FAA and depression, previous studies of PR-related variables and functioning have defined PR as a global psychological construct that is measured (i) on the basis of a single score from a self-report inventory, (ii) the presence/absence of a stress-related disorder, or (iii) a dichotomous classification of resilience on some other basis. Although they are common approaches to the definition of an independent variable, these methods suffer from two major limitations in terms of their ability to produce research findings of interest.

First, the dichotomisation of a variable such as PR reduces the actual variability in participants’ responses to a simple yes/no or high/low dichotomy, with attendant limitations upon the available statistical procedures that may be used to analyse such data [40]. Simply put, if (for example) the mean score on PR was used to dichotomise participants, someone who scores very low on PR is classified in the same category as another participant who scores just below average on PR, whereas these participants represent quite different degrees of PR. One method of overcoming this limitation is to apply correlational/regression procedures to PR data and their association with neurophysiological data.

Second, when treated as a unitary construct (as has been performed in all studies of the neurophysiological correlates of PR to date), the multifactorial nature of PR-related behaviours is ignored. For example, one of the most commonly used scales to measure PR is the 25-item Connor–Davidson Resilience Scale (CDRISC) [41]. The CDRISC was reported by its authors to possess five factors/components, each of which describes a cluster of PR-related behaviours, including (1) personal competence, high standards, and tenacity; (2) trust in one’s instincts, tolerance of negative affect, and strengthening effects of stress; (3) positive acceptance of change, and secure relationships; (4) control; (5) spiritual influences. Clearly, some of these factors are more likely to be associated with the BAS or the BIS than others, and so they hold relevance to further understanding of the ways that the BIS and BAS may interact with PR to influence vulnerability to depression. Although the factor structure of a scale will likely vary with the sample/population studied [42], no previous studies of PR and neurophysiological phenomena have examined the factor structure of the CDRISC within their samples, referring instead to the total score or a dichotomous classification of PR based upon CDRISC score. As such, the PR-related behaviours that act to produce the overall benefits of PR have not been described or examined for their respective neurophysiological correlates, thus limiting understanding of how PR itself occurs within the brain.

Thus, examination of PR at the factor/component/behaviour level is important because the five factors described by the CDRISC authors represent quite different strategies for dealing with stressful events. That is, trusting one’s own instincts is diametrically opposed to spiritual influences, and having a sense of control over events is a different strategy to positively accepting change. These different underlying components of the CDRISC may represent different neurophysiological pathways to PR-related behaviour, perhaps also linked with the BAS or BIS; coalescing them all into a single PR total score prevents more precise consideration of the nature of PR, PR-related behaviour, and their associations with neurophysiological variables.

Therefore, to further investigate the neurophysiological substrates of PR and to test the relevance of the FAA model of approach/withdrawal behaviour (i.e., the BAS vs. the BIS) to PR, this study examined the association between PR and FAA at the global PR level (i.e., CDRISC full-scale score), and also for the components of PR (i.e., clusters of behaviours underlying the CDRISC full-scale score). These components of PR were defined in two ways: first, by the five factors reported by the CDRISC authors [41], and second, by the factor structure found for the CDRISC within the current sample. Further, although most of the previous studies have used community samples, this study added a comparison between depressed and non-depressed subgroups from within a community to the analysis of total sample data in order to detect any difference in the PR-FAA association that might be influenced by depressive status. On the basis of the previous literature, it was hypothesised that there would be a significant association between FAA and PR at the total score level, but no directional hypotheses could be advanced for the association between FAA and the components of PR due to the lack of previous research on that issue.

## 2. Methods

### 2.1. Participants

The sample was drawn from volunteers aged 18 years or more (*M* age = 32.53 yr, SD = 14.13 yr) recruited from the New England region of New South Wales, Australia, via an advertisement for participants for “a study into how you think”. There were 54 males and 46 females aged between 18 and 75 years, with the age range breakdown of 18–30 yr: 54.5% males, 62.5% females; 35–50 yr: 29.6% males, 25.0% females; and 51 yr and over: 15.8% males, 12.5% females. None of these participants had a previous medical history of severe physical brain injury, brain surgery, or history of epilepsy, seizure disorder, or claustrophobia (EEG data were collected in a small booth). Because 61% to 70% of left-handed people also have left hemispheric dominance [43,44], selection did not include handedness.

### 2.2. Psychological Scales

PR was measured by the Connor Davidson Resilience Scale (CDRISC) [41], which consists of 25 items such as “I like a challenge”, “When things look hopeless I do not give up”, “I bounce back after illness or hardship”, and “I am able to adapt to change” [41]. Responses are given on a 5-point scale of “Not true at all” (0), “Rarely true” (1), “Sometimes true” (2), “Often true” (3), and “True nearly all of the time” (4) for how the respondent felt over the past month. This produces a total score between 0 and 100, with higher scores indicating greater resilience. Scores on the CDRISC are significantly directly correlated (.83) with total scores on the Kobasa Hardiness Measure and negatively correlated with total scores on the Perceived Stress Scale (−.76). Internal consistency (Cronbach alpha = .89) and test–retest reliability = .87 [41] are also sound. The five factors reported for the CDRISC by the scale authors were used, as well as the CDRISC total score and the factors found in the current sample.

Participants’ depressive status was measured by the Zung Self-Rating Depression Scale (SDS) [45], composed of ten positively-worded and ten negatively-worded questions about symptoms of Major Depressive Disorder [46]. Participants indicate the frequency of their experiences of the 20 SDS items during the last two weeks as “None or a little of the time” (score = 1), “Some of the time” (2), “Good part of the time” (3), or “Most or all of the time” (4). The SDS produces total scores from 20 to 80 [45,47], with 40 or above indicative of “clinically significant depression” [47] (p. 335). The SDS has split-half reliability of .81, [45], .79 [48] and .94 [49], with an internal consistency (alpha) of .88 for depressed patients and .93 for non-depressed patients [50].

### 2.3. EEG Data

EEG data were collected from a 40-channel *Quik*-*Cap*, with a focus on five pairs of frontal sites (FP1:FP2, F3:F4, F7:F8, FT7:FT8, FC3:FC4), using the average of the twin mastoids (A1 + A2) as the recording reference. A *Nuamps* digital EEG amplifier (Compumedics USA Ltd., El Paso, TX, USA) was used to collect continuous EEG measurements during 3-min Eyes Opened and 3-min Eyes Closed resting conditions. EOG data were recorded using electrodes at VEOR and HEOL below the right eye, and all dropdown electrode sites were cleaned with an alcohol swab and *Nuprep* gel so that all electrode impedances were < 5 KΩ. Participants were seated in an experimental booth while their EEG data were collected. EEG signals were recorded using the *Curry 7* software at a sampling rate of 1 KHz, with a bandpass of DC to 250 Hz.

Data were re-referenced to a common average, then processed using a low filter (high pass), frequency of 1 Hz and a slope of 2 Hz; a high filter (low pass) with frequency of 30 Hz and a slope of 8 Hz; a notch filter of 50 Hz (Harmonics, San Jose, CA, USA) with a slope of 1.5 Hz; and a band stop filter of frequency of 50 Hz (Harmonics) with a width of 10 Hz and slope of 5 Hz. A Hann window with a 10% width to prevent data loss was used to filter the data, which were then visually examined to identify artefacts (eye movements, muscle movements, electrode pop, etc.), all of which were removed from the data record. Bad block and eye blink detection (using the magnitude of eye blink deflections as a set threshold criterion to detect artefacts) was undertaken by three automated methods (Subtraction, Covariance and Principal Component Analysis) to produce clean EEG data.

Back-to-back epochs of 2 s duration were then created from the cleaned EEG data. Most participants had over 90% usable artefact-free epochs for both Eyes Opened and Eyes Closed conditions. Spectral analysis was performed on the generated epochs (for both conditions for each participant) with a Fast Fourier Transformation (FFT) to calculate the power spectra. The power values obtained from FFT were averaged across the 2-s EEG epochs. From this process, the total FAA power within the alpha (8–13 Hz) frequency range was obtained for each participant by subtracting the left hemisphere site alpha values from the right hemisphere site alpha values (e.g., FP2-FP1) to produce five sets of FAA data. These data were extracted and transferred to MATLAB, EEGLAB, and SPSS to calculate the correlations between CDRISC total score, factor scores, and FAA. Data from EEG sites were analysed using source localisation with *eLORETA* [51,52], which provides a high-density mapping of brain regions according to comparative electrical power recorded from EEG electrodes. This process was used for statistical and visual analysis of comparative alpha activity across brain regions, with a focus on the 10 frontal sites listed above.

There is some discussion in the EEG literature regarding the relative value of alpha wave data from eyes open versus eyes closed conditions, with evidence of differing topography and power values across these two conditions [53,54], principally that the eyes closed resting state represents lower arousal than the eyes open resting state [55]. To eliminate possible bias in results, both eyes open and eyes closed data were collected and analysed separately when testing the correlations between FAA and CDRISC factors.

### 2.4. Procedure

Participants read an Explanatory Statement and signed a Consent Form a background questionnaire (age, sex) and completed the CDRISC and SDS. Participants’ scalps were then prepared, and the electrode cap was fitted. Headphones were placed on participants so as to minimise the effect of external stimuli when instructions were given. Following 15 min of sitting still (adaptation), the audio-recorded experimental protocol (3 min Eyes Open, 3 min Eyes Closed) was presented via headphones to ensure consistency across participants. Ethics approval was received from the Human Research Ethics Committee of the University of New England, Australia (Approval No. HE14-051).

### 2.5. Statistical Analysis

Mean, standard deviation, standard error, and normality data were produced for the CDRISC and SDS. Internal consistency for the CDRISC and SDS were determined via Cronbach’s alpha. EEG data were tested for normality and found to be skewed. Although this has sometimes been dealt with by log transformation, there are inherent drawbacks to applying that process because it can hinder the interpretation of data, resulting in the recommendation that researchers should instead use the untransformed data with statistical procedures that are robust to non-normality [56]; in this case, that was Spearman’s Rank Order correlation. An additional advantage of Spearman correlations is that they also deal with any non-normality in other data, such as the CDRISC and SDS, whereas the Pearson correlation assumes that any variables being examined “follow a bivariate normal distribution in the population from which they were sampled” [57] (p. 1764). Psychological data (such as PR, depression, and EEG spectral power) are pervasively non-normal [58]. ANOVA and MANOVA, however, are robust to the confounding effects of non-normality [42].

Factor analysis was undertaken to identify the components of the CDRISC in this sample. The five original CDRISC factors were calculated from the raw scores on relevant items as stipulated by the CDRISC authors [41], and the mean for each factor was calculated to avoid confounds arising from differing numbers of CDRISC items within factors; the same procedure was followed for the factors derived from the current sample. Correlation coefficients were derived for the associations between the CDRISC total score and its factors and SDS scores and each of the five sets of FAA data. The equivalent regression analyses (CDRISC scores and alpha band power) were also calculated using source localisation data from *eLORET* [51] in order to examine the location of the associations between FAA and CDRISC factors at a more precise level throughout the frontal grey matter.

There is some disagreement regarding the need to make automatic corrections to alpha values when undertaking multiple tests of association [59], principally because reducing Type I error rates also increases Type II error rates [60], but also because such corrections may have “little effect on conclusions” [61] (p. 121). Consequently, it has been recommended that researchers “select a primary outcome measure or use a global assessment measure, rather than adjusting the *p*-value” before undertaking significance testing [62] (p. 1). Therefore, the present research followed this advice so that results were considered to be of meaningful value if they either met the standard *p* < .05 criterion or were of sufficient strength to qualify under Cohen’s [63] definition of a medium strength effect size (i.e., a correlation ≥ .3) [64].

## 3. Results

### 3.1. Data

Table 1 shows the descriptive data for the CDRISC and SDS. The 5% trimmed means were very close to the actual means, suggesting that there were negligible effects from outliers, although skewness was present. Internal consistency (Cronbach’s alpha) was satisfactory (>.85) for both the CDRISC and the SDS. There was no significant correlation between the sex of participants and CDRISC total score *ρ* = .091, *p* = .366, or SDS total score *ρ* = .022, *p* = .829, or between the age of participants and CDRISC *ρ* = .060, *p* = .553 or SDS *ρ* = .055, *p* = .584, allowing the data to be analysed without this potential confound.

As expected, CDRISC total score was significantly inversely correlated with SDS total score *ρ* = −.744, *p* < .001 (a large effect size) [63]. By applying the cutoff for “clinically significant depression” recommended by Zung [47] (p. 335), 33 participants (14 males) were classified into that category, and 67 (32 males) were classified as not clinically depressed. There was no significant difference in the ages of these two depression subgroups *F*(1,99) = .447, *p* = .506, but the clinically depressed sample had significantly higher SDS scores (*M* = 50.393, SD = 7.432) and significantly lower CDRISC (*M* = 78.636, SD = 15.093) scores than the non-clinically depressed participants (SDS *M* = 29.955, SD = 4.828: *F*(1,99) = 273,729, *p* < .001, η*p*^2^ = .736; CDRISC *M* = 99.343, SD = 10.409: *F* = 64.336, *p* < .001, η*p*^2^ = .396), both of which were large effect sizes [63].

### 3.2. PR and Depression

#### Total Sample

The sample means (SD) of the five factors of the CDRISC described by the scale The sample means (SD) of the five factors of the CDRISC described by the scale authors [41] are shown in the upper section of Table 2. To ensure that the consideration of the CDRISC components was comprehensive for this sample, these data were augmented by those from a factor analysis of the 25 CDRISC items for the 100 participants in this study. It has been argued that the key criteria for ensuring adequate sample size for factor analysis are the presence of many inter-item correlations of at least .3 [42], a Kaiser-Meyer-Olkin measure of sampling adequacy [65] of at least .6, and a significant Bartlett’s test of sphericity [66]. The CDRISC data from this study met these criteria (K-M-O measure = .913; Bartlett’s test = *p* < .001). By reference to eigenvalues > 1.0, the scree plot, and confirmed by parallel analysis, a two-factor solution was obtained. It should be noted that Connor and Davidson [41] reported only the use of eigenvalue data when identifying their factor structure, and the criteria used here are more stringent, perhaps accounting for the difference in factor structure, as well as the variability in factor structure that may occur when using alternative samples [42]. From this process, a two-factor solution emerged. Factor 1, titled *General resilience*, was very large, including all CDRISC items except items 3, 9, and 20, and accounting for 44.925% of the variance; Factor 2 (items 3 “Sometimes fate or God can help me”; 9 “Things happen for a reason”; and 20 “I have to act on a hunch”) was named *Reliance on others* and accounted for a further 7.915% of the variance. These factors were only moderately correlated (*r* = .218), suggestive of orthogonality, and allowing either Varimax or Oblimin rotation to be used; both of these procedures produced identical simple solutions. The means (SD) for these two factors are shown in the lowest two rows of Table 2. Because all of these seven factors were composed of different numbers of CDRISC items, the Factor means are also shown in the third column of Table 2 to allow for meaningful comparisons across all these factors, which were reasonably similar to each other. The fourth column of Table 2 shows the Spearman correlations between each of the seven CDRISC factors and SDS total score, reflecting the expected overall inverse correlations between PR and depression, with the exception of the original factor 1 *representing Spiritual influences*, and the current sample factor 2 *Reliance on others.*

### 3.3. Depressed vs. Not Depressed Subgroups

These results are relevant to the total sample but do not provide any insights into the scores of the clinically depressed versus not-clinically depressed subgroups, which may assist in understanding the associations between CDRISC factors, depression, and FAA. As a first step in that analysis, Table 3 presents the Spearman correlation coefficients between CDRISC total score and factors and SDS total score for the clinically depressed and not clinically depressed subgroups. By applying the criterion for meaningful results that was stated in the Methods (i.e., *p* < .05 or *r* ≥ .3), four of the five original CDRISC factors and one of the two factors found in the current study had significant inverse associations with total SDS for both the depressed and non-depressed subgroups. The fifth original CDRISC factor (not significantly associated with SDS score) contained two CDRISC items (item 3: *Sometimes fate or God can help;* item 9: *Things happen for a reason*). These two CDRISC items were also included in the second factor from the current study, plus item 20 (*I have to act on a hunch*). These PR-related behaviours do not appear to assist in reducing the likelihood of developing depression.

### 3.4. PR and FAA

The results of the data analysis aimed at understanding the associations between FAA data and the total CDRISC and its factors are presented in Table 4 for the not clinically depressed subgroup and the clinically depressed participants. Only those correlation coefficients that reached the criterion for meaningful results are shown in Table 4. The most obvious result from these analyses is the general difference in the direction of the correlation coefficient between the CDRISC factors and FAA for the not clinically depressed (column 4) versus the clinically depressed (columns 3 and 5) subgroups for CDRISC Original Factor 4. Those participants whose SDS scores indicated that they were suffering from clinically significant depression had uniformly *direct* correlation coefficients between the CDRISC scores and their FAA data, but those participants whose SDS scores did not reach the level of severity stipulated by Zung [47] had uniformly *inverse* correlation coefficients between their CDRISC scores and FAA data on that component of the CDRISC that measured participants’ *control.*

To understand the meaning behind these inverse vs. direct correlations, because the CDRISC scores are always positive, an inverse correlation could only occur if the FAA value was negative. The FAA values were obtained by subtracting the left hemisphere site alpha values from the right hemisphere site alpha values (e.g., FP2-FP1) so that a negative value indicated that the left hemisphere had higher alpha activity than the right hemisphere, represented using the statement: Lα > Rα. When submitted to Spearman correlational analysis, this kind of relative brain site alpha activity produced inverse correlation coefficients between FAA and the CDRISC scores, as was found for the not clinically depressed subgroup. By contrast, the clinically depressed subgroup (whose correlation coefficients were all direct) had higher alpha activity in their right hemisphere than in their left hemisphere Rα > Lα.

Four of the five FAA pairs (i.e., FP2:FP1, F8:F7, FT8:FT7, FC4:FC3, but not F4:F3) showed meaningful correlations with some of the CDRISC factors. Although four of the five original CDRISC factors were found to have at least some meaningful correlations with some FAA data, whether from eyes-open or eyes-closed conditions for either or both of the not-depressed and depressed subgroups, those associations were not uniformly spread across the entire frontal region, but were indicative of specific frontal regional activity being associated with specific aspects of PR. This specificity is reflected in the different directions of the correlations for not-depressed versus depressed subgroups described above.

For the two-factor solution found in the current sample, factor 2 (Reliance on others) appears to be very similar to the original factor 5, both in terms of the absence of any significant correlations with the SDS scores (Table 3) and also in terms of the scalp sites that were associated with those factors (i.e., FP2:FP1; FT8:FT7) (Table 4). Current Factor 1 may be a combination of the first four Original CDRISC factors, both in terms of CDRISC items (i.e., all items except those in Current Factor 2) and also because of the significant correlation between its mean scores and the F8:F7 site, which was also observed for CDRISC Original factors 1, 2, and 4.

Finally, when considering these data at the FAA level, there were 10 meaningful correlations under the eyes open condition, but only two under the eyes closed condition, suggesting that the eyes open/closed variable does have some effect on the associations between FAA and CDRISC factors. That effect is no doubt due to the alpha wave activity generated rather than the CDRISC factors and will be discussed in more detail below.

### 3.5. Location of PR Component Alpha Activity

These data regarding FAA are informative at a gross scalp region level but do not provide insights into the specific areas of the brain that were associated with CDRISC factor scores. To examine those associations, the regression analyses on the relationship between CDRISC scores and alpha band power (8–13 Hz) were performed using source localisation data via *eLORETA* [51]. Whilst there were no individual voxels that reached statistical significance (*p* < .05), the results showed broadly consistent results indicating stronger alpha power in the frontal right hemisphere. To avoid needless repetition, only the relevant results from analyses with significant Spearman correlations for original factor scores in the eyes-open condition from Table 4 are presented here.

**Figure 1:**
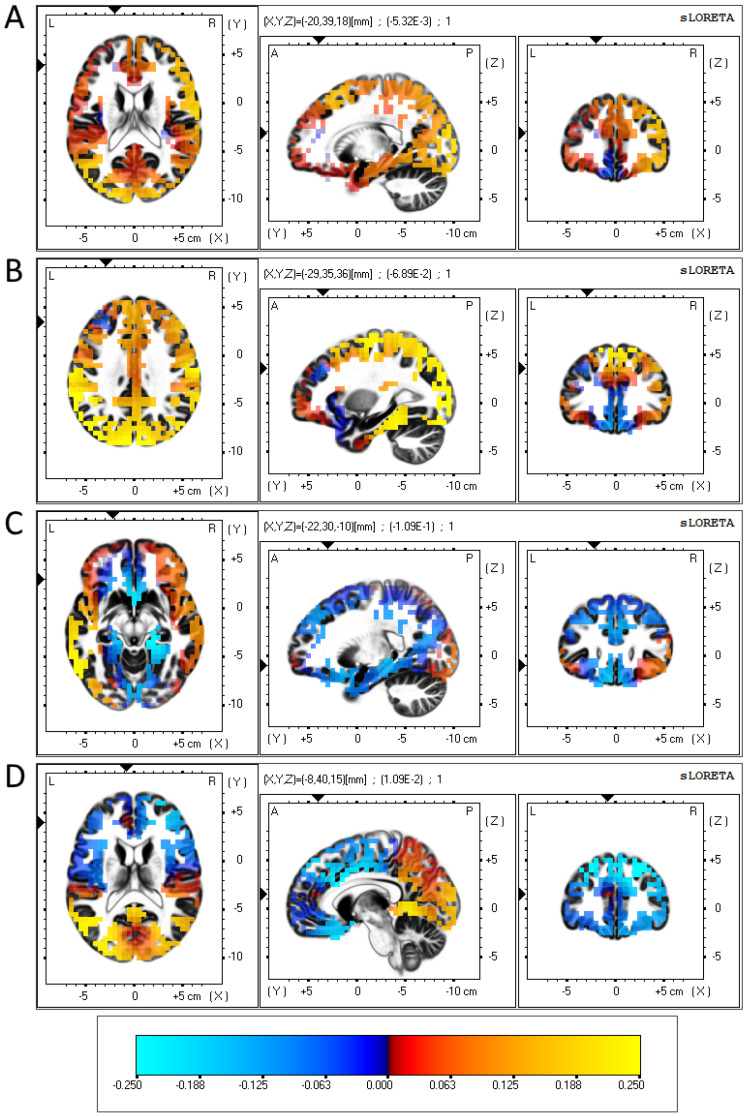
eLORETA-sourced regression analysis of changes in alpha band power predicted using CDRISC factor scores in the depressed sample.

CDRISC Original Factor 1: While the frontal right hemisphere showed broadly consistent increases in alpha power as the factor score increased, the frontal left hemisphere showed comparatively lower increases in alpha power, with a small decrease observed in the left medial frontal gyrus (Brodmann area (BA) 9); see Figure 1A.

CDRISC Original Factor 2: Similar to the results regarding factor 1, the frontal right hemisphere showed broadly consistent increases in alpha power as the factor score increased, while a localised fall in alpha power was observed in the left medial frontal gyrus (BA 9); see Figure 1B.

CDRISC Original Factor 4: Consistent rises and falls in regression coefficients were found across each frontal hemisphere, with the exception of a 4 cm strip of grey matter along the middle frontal gyrus (BA 11) and inferior frontal gyrus (BA 47), which produced a negative regression coefficient in the left hemisphere and a positive coefficient in the right hemisphere; see Figure 1C.

CDRISC Original Factor 5: This analysis showed a consistent negative relationship between factor 5 and alpha band power across both frontal hemispheres, with a localised positive regression coefficient in the left anterior cingulate (BA 32); see Figure 1D.

## 4. Discussion

By applying a non-dichotomous metric of total PR score from the CDRISC, plus an examination of the underlying components of that construct and instrument, several confirmatory and extending behavioural findings emerged from this study of the neurophysiological correlates of PR. First, the hypothesis that global PR would be inversely associated with depression was supported. Second, the finding that only Factors 1 to 4 of the CDRISC, and Factor 1 from the current sample, had significant inverse correlations with SDS score suggests that the CDRISC contains some items that are not, on the basis of these data, robust protectors against depression (i.e., those in Original Factor 5 and Current Factor 2). Generally speaking, when participants sought to rely on others to cope with stressful events, that strategy was not as effective in avoiding depression compared to taking some more active- and self-belief-based path to deal with stress. This lack of internal consistency in terms of PR’s effectiveness in helping participants avoid or reduce depression suggests that PR ought not to be considered a unitary construct but rather a set of (sometimes conflicting) components, at least in the CDRISC.

The neurophysiological aspects of PR were the major focus of this investigation, and the results reported above extend those previously found, such as Bae et al. [30] and Ma et al. [31], regarding the association between FAA and coping with stressors. Those studies did not include an examination of the role of PR and its components in their interaction with FAA and depression, nor an in-depth examination of brain sites where components of PR were active. Data pictured in both Figure 1A,B implicate reduced activity in the left medial prefrontal cortex (mPFC) as neural correlates of depression. Several studies have reported that increased levels of depression are associated with a decrease in the volume of grey matter in this region compared to the right mPFC [67,68], which is consistent with the BAS/BIS depression hypothesis. Relatedly, treatments such as transcranial magnetic stimulation commonly focus on stimulating the left (rather than the right) mPFC [69]. Additionally, the reduced alpha activity in the left inferior frontal gyrus (see Figure 1C) also corresponds with reports showing inter-hemispheric differences in activation at this section of the brain [37]. Due to the reciprocal connections the mPFC has with both the amygdala and the hippocampus, activity in the mPFC is strongly associated with mental functions involving memory and emotional processing, particularly those involving motivation, apathy and concentration [70]. These functions align well specifically with CDRISC Factors 1 and 2, so it may be the case that differences in FAA are associated with depression-based dysfunctions in the mPFC, where PR has not been effictive.

Figure 1C shows a lateralised difference in activity in the left middle and inferior frontal gyrus, both of which form up parts of the orbitofrontal cortex (OFC). Similar to the mPFC, people with depression are reported to have a lower volume of grey matter in the left OFC compared to control subjects [71], while decreases in left OFC activation (as well as increases in right OFC activation) have been associated with goal-specific dysfunctions in those with depression [72]. As the OFC has strong white matter connections with the hypothalamus, activation of the OFC is implicated with impulse control and self-regulation [70,73,74], which corresponds well with CDRISC Factor 4 (Control).

For Factor 5 (Figure 1D), the comparatively small effect size, plus the proximity of the two electrodes on which this was based (FP1, FP2), may have produced inconsistent left hemispheric dominance, reflecting the lack of a statistically significant inverse association with depression that was demonstrated using the other four CDRISC factors.

These initial findings are limited in scope but do provide a firm basis for considering the PR construct as composed of different components that are not all equally strongly inversely associated with depression. In fact, CDRISC Factor 5 does not fulfil this task significantly and may be questioned as to its justification within the overall PR construct. Secondly, the heterogeneity of the PR construct, as measured via the five components of the CDRISC, received initial support from the different neurocognitive manifestations shown in Figure 1, which argue for the presence of different neurocognitive processes. Although this might be conjectured on the basis of the CDRISC items within each of its factors, the FAA data collected here confirm that heterogeneity in a much firmer manner than previously and argue for a neurophysiological basis underlying the apparent behavioural content measured using different CDRISC factors. Overall, the different direction of the correlations between FAA and CDRISC factors that were found for the depressed versus not-depressed participants was a major finding here and suggests that PR manifests itself in different hemispheres depending upon whether it is acting successfully (i.e., in participants who are not depressed) compared to those in which it has not proven to be efficacious in buffering against depression.

Limitations in this study included the focus on frontal alpha wave activity. Although this was justified because the present study was aimed at testing the FAA-PR hypothesis on the basis of the previous literature regarding FAA depression, an extension of the data to include other brain regions and brain wave frequencies would enhance understanding of the neurobiological underpinnings of PR. The participants were all volunteers and, despite there being a reasonable proportion of participants who met the criteria for clinically significant depression, did not include a clinically diagnosed subsample, thus limiting the generalisation of these data to that subgroup. Data were cross-sectional, and no account can be given of fluctuations in the associations between FAA and CDRISC factors over time or under stressful conditions--these are promising fields for future research. Although not a limitation per se, the difference in results between eyes open and eyes closed conditions suggests the need to further investigate the effects of environmental visual stimulation upon alpha wave data in experiments such as this. As noted in the Methods, there is other evidence of differing power values and topography across these two conditions [53,54,55], and that finding was apparently replicated here.

## 5. Conclusions

Although PR was originally defined in psychological and behavioural terms, recent research has focussed on using EEG and MRI to define and describe the neurobiological processes that underlie resilient behaviour [20,75,76]. The data from the current study add to that *corpus* and extend it by providing a new understanding of the specific components of PR and how these may be associated with particular brain region activity. The major outcome of this study (i.e., that PR, as measured with the CDRISC, is neurophysiologically heterogeneous as well as in terms of the item content in its five factors) holds major implications for research and clinical settings where PR is usually assumed to be homogeneous.

## Figures and Tables

**Table 1:** Descriptive data for CDRISC ^1^ and SDS ^2^.

Scale	Mean	Standard Deviation	Standard Error	Range	5% Trimmed Mean	Cronbach Alpha
CDRISC	92.511	15.544	1.554	51–118	93.211	.930
SDS	36.700	11.256	1.125	21–60	36.133	.921

^1^ CDRISC = Connor–Davidson Resilience Scale; ^2^ SDS = Zung Self-rated Depression Scale.

**Table 2:** Sample means (SD), Factor means, and Spearman correlations with SDS ^1^ total score for CDRISC ^2^ original five factors plus two factors from the current sample.

CDRISC Factors	Sample Mean (SD)	Factor Mean	Correlation with SDS Total Score
Original Factor 1: *Personal standards, high competency, tenacity*	31.090 (5.648)	3.886	−.680 *
Original Factor 2: *Trust in one’s instincts, tolerance of negative affect, strengthening effects of stress*	23.860 (3.848)	3.409	−.615 *
Original Factor 3: *Positive acceptance of change, secure relationships*	19.950 (3.927)	3.990	−.740 *
Original Factor 4: *Control*	11.290 (2.629)	3.763	−.654 *
Original Factor 5: *Spiritual influences*	6.320 (2.436)	3.160	−.104 ^3^
Current Factor 1: *General resilience*	83.410 (14.704)	3.791	−.740 *
Current Factor 2: *Reliance on others*	9.100 (2.768)	3.000	−.080 ^4^

^1^ SDS = Zung Self-rated Depression Scale; ^2^ CDRISC = Connor–Davidson Resilience Scale; * *p* < .005; ^3^ *p* = .234; ^4^ *p* = .292.

**Table 3:** Spearman correlation coefficients between CDRISC ^1^ total score and factors with SDS ^2^ total score for not-clinically depressed versus clinically depressed subgroups.

Scale/Factor	Not Clinically Depressed (*n* = 67)		Clinically Depressed (*n* = 33)	
	*ρ*	*p*	*ρ*	*p*
CDRISC total score	−.545	<.001	−.488	.004
Original Factor 1	−.508	<.001	−.377	.031
Original Factor 2	−.333	.006	−.519	.003
Original Factor 3	−.530	<.001	−.507	.004
Original Factor 4	−.540	<.001	−.435	.011
Original Factor 5	−.072.	.561	−.230	.199
Current Factor 1	−.549	<.001	−.489	.004
Current Factor 2	−.045	.717	−.225	.207

^1^ Connor–Davidson Resilience Scale; ^2^ Zung Self-rating Depression Scale.

**Table 4:** Meaningful Spearman correlation coefficients (and *p* values) between CDRISC ^1^ factor scores and FAA ^2^ data for not-clinically depressed and clinically depressed participants under eyes open and eyes closed conditions.

	Eyes Open		Eyes Closed	
	Not-Clinically Depressed (*n* = 67)	Clinically Depressed (*n* = 33)	Not-Clinically Depressed (*n* = 67)	Clinically Depressed (*n* = 33)
Original Factor 1: *Personal standards, high competence, tenacity*		F8:F7; .353 (.042)		
Original Factor 2: *Trust in one’s instincts, tolerance of negative affect, and strengthening effects of stress*		F8:F7; .384 (.028)		
Original Factor 3: *Positive acceptance of change and secure relationships*				
Original Factor 4: *Control*		F8:F7; .511 (.002) FT8:FT7; .428 (.015)	FC4:FC3; −.272 (.026)	FT8:FT7; .323 (.067)
Original Factor 5: *Spiritual influences*		FP2:FP1; .407 (.019) F8:F7; .315 (.075) FT8:FT7; .329 (.062)		
Current Factor 1: *General resilience*		F8:F7; .404 (.020)		
Current Factor 2: *Reliance on others*		FP2:FP1; .311 (.018) FT8:FT7; .340 (.079)		

^1^ Connor–Davidson Resilience Scale; ^2^ Frontal Alpha Asymmetry.

## Data Availability

Data are available from the first author on reasonable request.
